# Biparametric MRI of the prostate radiomics model for prediction of pelvic lymph node metastasis in prostate cancers : a two-centre study

**DOI:** 10.1186/s12880-024-01372-8

**Published:** 2024-07-25

**Authors:** Chunxing Li, Jisu Hu, Zhiyuan Zhang, Chaogang Wei, Tong Chen, Ximing Wang, Yakang Dai, Junkang Shen

**Affiliations:** 1 Department of Radiology, The Second Affiliated Hospital of Soochow University, Suzhou, China; 2 Department of MRI Room, Yancheng First Hospital Affiliated Hospital of NanJing University Medical School, Yancheng, China; 3 Suzhou Institute of Biomedical Engineering and Technology, Chinese Academy of Sciences, Suzhou, China; 4 School of Medical Imaging, Biomedical Engineering, Xuzhou Medical University, Xuzhou, China; 5 Department of Radiology, The First Affiliated Hospital of Soochow University, Suzhou, China

**Keywords:** Magnetic resonance imaging, Radiomics, Prostate cancer, Pelvic metastatic lymph nodes

## Abstract

**Objectives:**

Exploring the value of adding correlation analysis (radiomic features (RFs) of pelvic metastatic lymph nodes and primary lesions) to screen RFs of primary lesions in the feature selection process of establishing prediction model.

**Methods:**

A total of 394 prostate cancer (PCa) patients (263 in the training group, 74 in the internal validation group and 57 in the external validation group) from two tertiary hospitals were included in the study. The cases with pelvic lymph node metastasis (PLNM) positive in the training group were diagnosed by biopsy or MRI with a short-axis diameter ≥ 1.5 cm, PLNM-negative cases in the training group and all cases in validation group were underwent both radical prostatectomy (RP) and extended pelvic lymph node dissection (ePLND). The RFs of PLNM-negative lesion and PLNM-positive tissues including primary lesions and their metastatic lymph nodes (MLNs) in the training group were extracted from T2WI and apparent diffusion coefficient (ADC) map to build the following two models by fivefold cross-validation: the lesion model, established according to the primary lesion RFs selected by t tests and absolute shrinkage and selection operator (LASSO); the lesion-correlation model, established according to the primary lesion RFs selected by Pearson correlation analysis (RFs of primary lesions and their MLNs, correlation coefficient > 0.9), t test and LASSO. Finally, we compared the performance of these two models in predicting PLNM.

**Results:**

The AUC and the DeLong test of AUC in the lesion model and lesion-correlation model were as follows: training groups (0.8053, 0.8466, *p* = 0.0002), internal validation group (0.7321, 0.8268, *p* = 0.0429), and external validation group (0.6445, 0.7874, *p* = 0.0431), respectively.

**Conclusion:**

The lesion-correlation model established by features of primary tumors correlated with MLNs has more advantages than the lesion model in predicting PLNM.

**Supplementary Information:**

The online version contains supplementary material available at 10.1186/s12880-024-01372-8.

## Background

Cancer stands out as one of the fatal diseases people are facing all the time, hence prognosis or survival of cancer patients has become the focus of current research [1]. Studies have shown that pelvic lymph node metastasis (PLNM) in prostate cancer (PCa) patients is closely related to distant metastasis and biochemical recurrence after curative treatment and influence the prognosis and survival of patients [2, 3]. Therefore, it is particularly important to accurately identify the PLNM of localized PCa in patients [4].

To evaluate whether a PCa patient has PLNM, clinical workers initially developed various prediction models based on the clinical and biopsy information, such as the Briganti or MSKCC nomograms or Partin Tables [5–7]. With the emergence of prostate magnetic resonance imaging (MRI) [8, 9], predictive factors based on MRI information have been favoured by researchers, such as the MRI-based qualitative characteristics of primary PCa lesions (measured tumour size, extraprostatic extension and seminal vesicle invasion) [10, 11]. MRI-targeted biopsies have been used as a substitute for systematic biopsy to obtain a more accurate Gleason score [12], and the MRI-based quantitative RFs and deep features of the primary lesions are extracted by radiomics and deep learning methods [13]. Current research is focused on mining primary lesion features that are conducive to predicting PLNM and on improving the performance of using primary lesion features in predicting PLNM. It is not known whether the RFs of pelvic metastatic lymph nodes (MLNs) can help improve the performance of the radiomics model of primary lesions in predicting PLNM in PCa.

Currently, extended pelvic lymph node dissection(ePLND) is the gold standard for the diagnosis of PLNM in PCa. According to this standard, imaging findings of pelvic LNs with an oval short axis diameter of ≥ 1 cm or a round short axis diameter of ≥ 0.8 cm are considered to indicate malignant LNs [10, 14–16]. Using this short axis diameter criteria, studies have shown that the pooled sensitivity of conventional MRI is 39% and the pooled specificity is 82% [17], and the pooled sensitivity of diffusion-weighted imaging (DWI) is 41% and the pooled specificity is 94% [18]. MRI evaluation of PLNM has low sensitivity and high specificity. A meta-analysis has previously shown that with improvement of the diagnostic criteria, that is, the increase in the positive lymph node threshold, there is an increase in the specificity of MRI diagnosis of MLNs. When the threshold is greater than or equal to 1.5 cm, the specificity is very high (up to 98 − 100%) [19]. Therefore, for patients with biopsy-confirmed PCa, when biparametric MRI (bpMRI) comprising T2-weighted imaging (T2WI) and DWI showed that the LNs (short axis diameter ≥ 1.5 cm) were round or oval or that the hilum of the lymph node had disappeared, or obviously high signal on high b-value DWI, the author had reason to believe that the abnormal LNs were MLNs.

Cancer lesions of pelvic LNs are metastatic from primary lesions, and thus, metastatic tissue in pelvic LNs should have the metastatic characteristics of the primary lesions. Their RFs should be closely related to those representing the metastatic characteristics of the primary lesions. With the help of the RFs of pelvic MLNs, it would be an interesting research direction to screen for more reasonable RFs of primary lesions and thus build a better predictive model. Here, we conducted this study to extract the RFs of pelvic metastatic LNs and primary lesions respectively, and performed correlation analysis to obtain RFs of primary tumors correlated with metastatic lymph nodes. Finally, we established an radiomics model to check its performance in predicting PLNM.

## Patients and methods

### Patients

A total of 394 PCa patients from two tertiary care centers, Center 1 and Center 2, were included in the study. Patients from Center 1 were enrolled between May 2016 and July 2021, while those from Center 2 were enrolled between January 2019 and May 2020. The training group, which consisted of 93 PLNM-positive and 170 PLNM-negative PCa patients, and the internal validation group, which included 19 PLNM-positive and 55 PLNM-negative PCa patients, were both from Center (1) The external validation group, comprising 14 PLNM-positive and 43 PLNM-negative PCa patients, was from Center (2) This retrospective study was approved by the institutional ethics committees of the two tertiary care centers. The requirement for informed patient consent was waived.

### Inclusion of positive cases in the training group

**Fig. 1:**
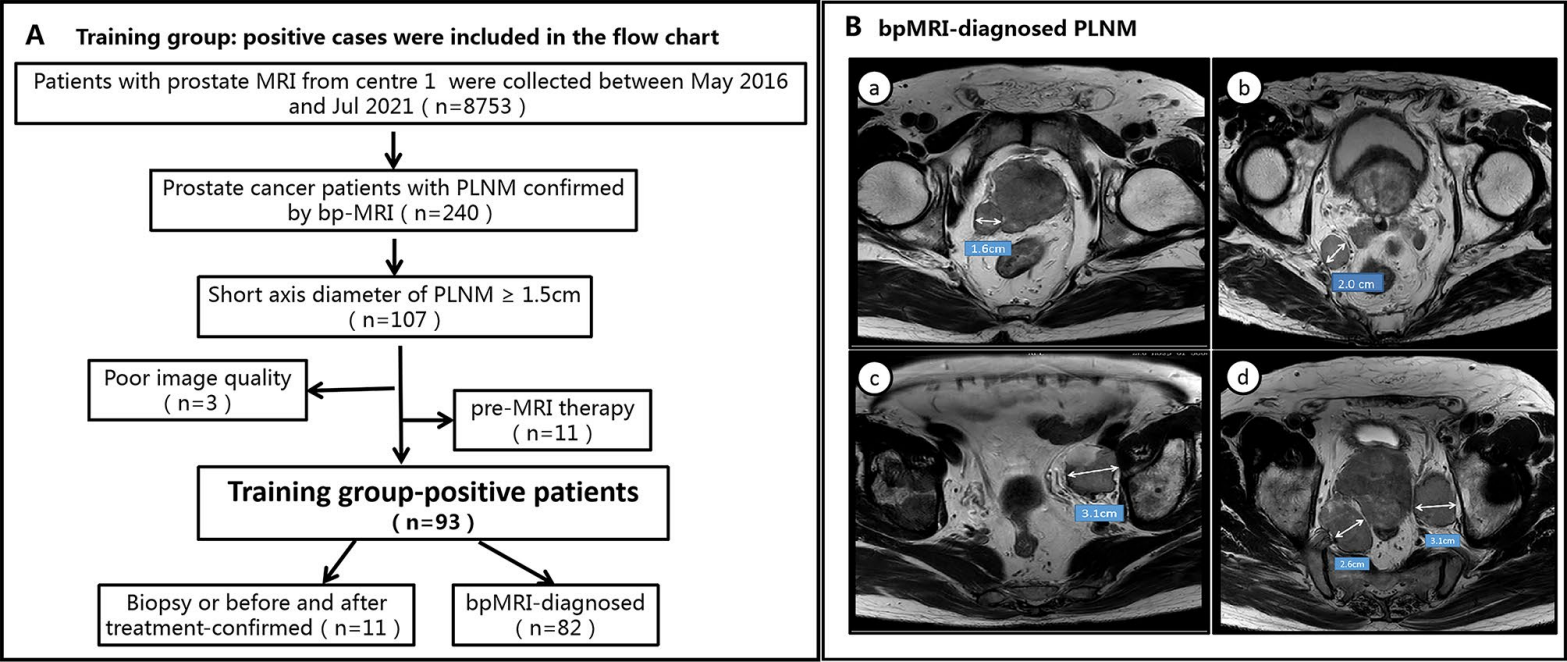
**A** shows the inclusion and exclusion criteria of positive patients in the training group. **B** shows bpMRI-diagnosed PLNM: four patients (a, b, c, d) with PLNM shown on T2WI (DWI was not added due to length) were included according to the criteria of pelvic metastasis LNs specified herein; a-d representative images showed that abnormal LNs with enlarged morphology (short axis diameter > 1.5 cm) and heterogeneous signals could be seen in the right paraprostatic area, the mesorectum, left iliac vessels and bilateral iliac vessels, respectively. RP: radical prostatectomy; PLNM = pelvic lymph node metastasis; bpMRI = biparametric MRI (T2WI and DWI)

All PCa patients enrolled in the training group were diagnosed as PLNM-positive by bpMRI. Drawing on previous studies that utilized MRI or CT to detect suspected pelvic LNs ≥ 1.5 cm, which were subsequently confirmed as MLNs after surgery [20–23], the present study also validated the diagnoses of 11 PLNM-positive patients in the training group. These included 4 patients diagnosed by biopsy and 7 diagnosed through pre- and post-treatment comparisons by MRI. For suspicious LNs diagnosed by bpMRI and with a short axis diameter ≥ 1.5 cm, the biopsy results and the decreased sizes of lymph nodes pre- and post-treatment were considered confirmation of metastasis. Therefore, we stipulated that metastatic LNs met the following requirements: (i) lymph nodes were round or oval, the hilum of the lymph node had disappeared or there was a heterogeneous signal intensity on high-resolution T2WI; (ii) high b-value DWI showed obvious high signal intensity, and apparent diffusion coefficient (ADC) map showed obvious low signal intensity; and (iii)the diameter of the short axis of abnormal LNs diagnosed by bpMRI was ≥ 1.5 cm. A flow diagram of the patient selection process with the inclusion and exclusion criteria is provided in Fig. 1(A, B).

### Inclusion criteria of the training group (negative cases) and validation groups

**Fig. 2:**
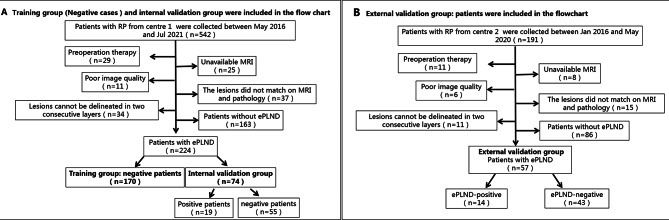
**A** shows the inclusion and exclusion criteria of the training group (negative cases) and the internal validation group. **B** shows the inclusion and exclusion criteria of the external validation group. ePLND = extended pelvic lymph node dissection

The inclusion criteria of the training group (negative cases) and validation groups were patients who underwent both RP and ePLND. None of the patients had a history of previous surgery, radiotherapy or adjuvant therapies for PCa before their prostate MRI. The inclusion/exclusion criteria are shown in detail in Fig. 2 (A, B).

### MRI protocols and sequence selection

All patients underwent 3.0T multiparametric MRI(mpMRI)or bpMRI scans with a pelvic phased-array coil, and the scan protocols and sequence were in accordance with Prostate Imaging Reporting and Data System version 2.0 or 2.1 (including T2WI, DWI, and/or dynamic contrast-enhanced (DCE) imaging). ADC maps were calculated on a designated workstation with b values of 100 and 1000 s/mm2. The manufacturers and models of the MR machines in the two hospitals are as follows: at centre 1, Philips Ingenia, Best, Netherlands; and at centre 2: Siemens, skyra, Erlangen, Germany.

Since recent studies have shown that bpMRI may serve as a faster, cheaper, gadolinium-free alternative to mpMR [24]. Therefore, in this study we selected bpMRI as the research sequence. Additionally, a study has previously shown that compared with high b-value DWI, using an ADC map for RFs extraction could provide superior stability [25]. Finally, we selected high-resolution T2WI and ADC maps for RFs extraction. The scan parameters and machine models of the hospitals are listed in Supplementary 1.

### Reference standard for pathology

All patients underwent a TRUS-guided 12-core systematic biopsy within 1 week after the prostate MRI scans. RP with ePLND was performed within 4 weeks after the prostate MRI scans. According to the ISUP 2005 and 2014 recommendations [26, 27], the histopathology of the specimens was assessed by experienced pathologists at the two hospitals.

In the evaluation of ePLNDs, the numbers and locations of normal LNs and MLNs were determined. According to the EAU risk group [28], RP with ePLND was performed in moderate-risk (unfavourable prognoses) and high-risk patients. Local LN dissection included dissection of the external iliac and obturator LNs. Expanded LN dissection included dissection of the obturator, external iliac, internal iliac, presacral and common iliac LNs. Four cases of biopsy-confirmed pelvic MLNs in the training group were found in the external iliac LN group in this study.

### Segmentation of cancer lesions on T2WI and ADC maps

The T2WIs (DICOM format) of all enrolled patients were imported into ITK Snap software (v3.8; http://www.itksnap.org/pmwiki/pmwiki.php). The ROIs of the primary lesions and their metastatic LNs were manually delineated by slicing along the contour of the cancer lesion and avoiding any calcification, bleeding, cysts and other structures present in the primary lesion and MLNs. The volume of interest (VOI-1) of the primary lesion and VOI-2 of MLNs were finally obtained by multilayer fusion in ITK Snap. The ADC images were registered and resampled to the corresponding T2W images, and the annotations of the ADC images were copied from those of the T2W images.

### Radiomic feature extraction, selection and construction

**Fig. 3:**
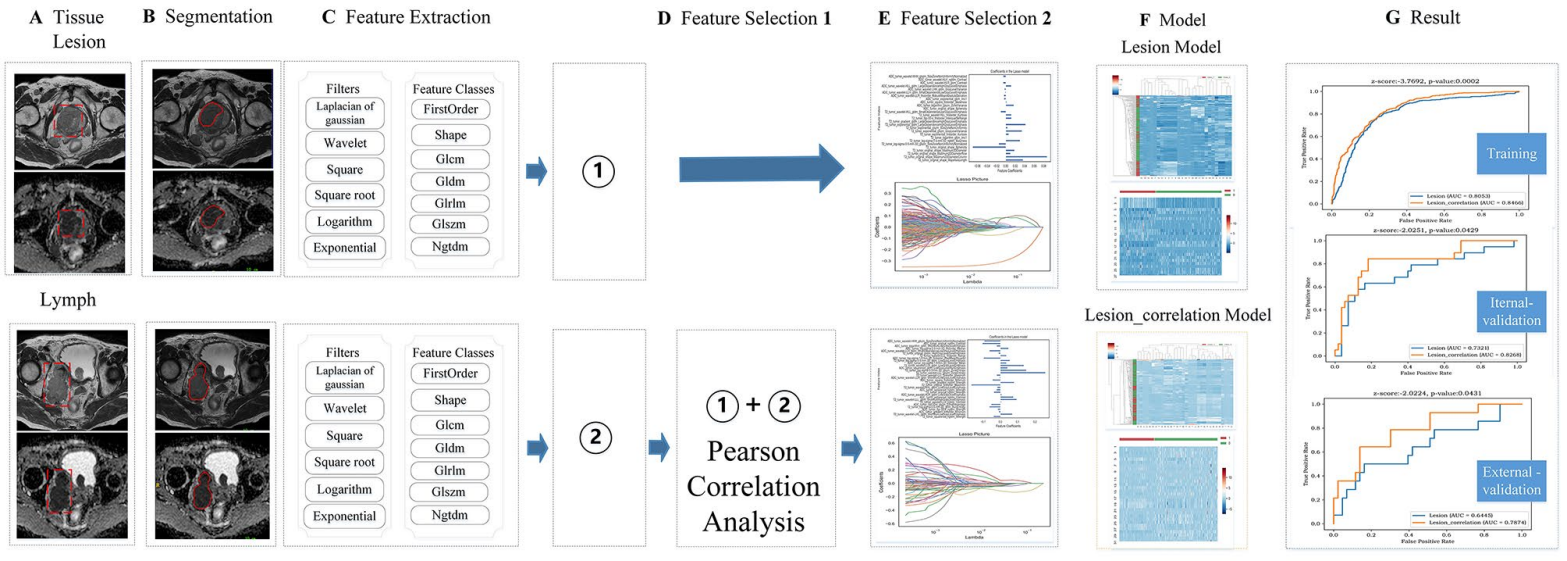
shows the radiomics workflow in this study. **A** and **B** show the primary lesion and its metastatic pelvic LNs and the ROI on T2WI and ADC map. **C** shows the types of extraction of RFs of the primary lesion and its pelvic MLNs, respectively. **D** shows two pathways: ① shows that the extracted primary lesion features have no feature selection in the D link. ② shows that the extracted RFs of the primary lesion and its MLNs were performed by a Pearson correlation analysis for feature selection, and the primary lesion RFs were selected by Pearson correlation analysis. E shows that two pathways were performed by the LASSO algorithm for feature selection. F shows the establishment of the Lesion model and Lesion-correlation model. G shows the performance comparison of the two models in the training group and the internal and external validation groups. LASSO = absolute shrinkage and selection operator

The RFs of PLNM-negative lesion and PLNM-positive including primary lesions and their MLNs in the training group were extracted using PyRadiomics (version 2.1.0;https://pyradiomics.readthedocs.io/en/2.1.0/), On this basis, this study refers to a new machine learning-based user-friendly software platform for automatic RFs extraction, feature selection, model building, model training and analysis [29] (Fig. 3). This study conducted a self-assessment of our work using the CLEAR checklist [30]and METRICS [31], which were submitted as Supplementary 2 and Supplementary 3.

We extracted features from the original images of the lesion area and metastatic LN area and from the images that were transformed by log (Sigma: [0.5,1,1.5]), logarithm, square, square root, exponential, gradient, lbp3d and wavelet (start_level: 1, level: 1, wavelet: Haar). Seven types of RFs were derived from the T2WI and ADC maps: first order, shape, gray level co-occurrence matrix (GLCM), gray level run length matrix (GLRLM), gray level size zone matrix (GLSZM), neighbourhood gray tone difference matrix (NGTDM), and gray level dependence matrix (GLDM). The following settings for feature extraction were used: b-spline interpolator, resampled pixel spacing of [1], pad distance of 10, bin width of 25, yoxel array shift of 300 and normalize scale of 100.

Prior to the feature selection, all RFs were normalized to zero mean and unit variance by standardization to prevent features with a large numerical range from dominating features with a small numerical range. Using the Pearson correlation analysis algorithm, the t test and lesast absolute shrinkage and selection operator (LASSO) algorithm were applied to screen for the best RFs for predicting PLNM status. The inter- and intraclass correlation coefficients (ICCs) between the extracted features of radiologists A (first time) and B were 0.7513–0.9799, and those of radiologist A (twice) were 0.7544–0.9748, which showed that the two signatures had good consistency. The Synthetic Minority Over-sampling Technique (SMOTE) was used to augment the RFs in the minority group of the training set [32].

The support vector machine (SVM) classifier was used to establish the radiomics model. When training the model, fivefold cross-validation was adopted and repeated 5 times to select the best training parameters and build the following two models: the lesion model, established according to the RFs selected directly by the t test and LASSO algorithm after extracting the primary lesion features; and the lesion-correlation model, established by first performing Pearson correlation analysis (correlation coefficient > 0.9) between the primary lesion features and MLN features, retaining only some of the primary lesion features that were closely correlated with MLN features, and second performing the t test and LASSO algorithm.

### Statistical analysis

Patient age, PSA, pathological information and MRI stage were statistically analyzed by Statistical Package for Social Science (SPSS, version 21.0, https://www.ibm.com/cn-zh/ analytics/spss-statistics-software). A two-sample t test was used to compare the differences in age and PSA level, while chi-square or Fisher exact tests, as appropriate, were used to compare the differences in categorical variables (MRI-based stage, pathological information). Intra- and interclass correlation coefficients (ICCs) were assessed using the kappa test. The performance of the lesion model and lesion-correlation model was assessed with receiver operating characteristic (ROC) curves in the training group and internal and external validation groups. The area under the curve (AUC), accuracy, sensitivity and specificity were calculated. The differences in the AUC values of the two models were assessed by the DeLong test. There was a significant difference in the bilateral test (*P* < 0.05).

## Results

### Clinical characteristics

A total of 394 PCa patients were ultimately enrolled in our study. The patients with PLNM accounted for 93/263 (35.4%), 19/74 (26.7%) and 14/57 (24.6%) in the training, internal validation and external validation groups, respectively. In the training group, 93 positive patients had 163 metastatic LNs (bpMRI-diagnosed and short axis diameter ≥ 1.5 cm), and the average diameter of the metastatic LNs was 2.3 cm. The statistical results for the clinical, pathological and MRI data of each group are described in detail in the list (Table 1).

**Table 1:** The characteristics of the patients in the training and validation groups

Characteristic	Training group			Internal-validation group			External-validation group		
	PLNM (+)	PLNM (-)	*p*	PLNM (+)	PLNM (-)	*p*	PLNM (+)	PLNM (-)	*p*
	*n*=93	*n*=170		*n*=19	*n*=55		*n*=14	*n*=43	
**age**,** mean**	73.7204	70.8235	0.002*	71.25	69.7234	0.373	66.5455	68.625	0.412
**PSA(ng/mL)**,** mean**	95.6	33.4	0.000*	63.4	35.7	0.000*	70.6	32.3	0.000*
**MRI-based stage**,** No. (%)**			0.000*			0.000*			0.000*
**T2**	2/93 (2.1)	130/170 (76.5)		3/19 (15.8)	29/55 (52.7)		5/14(35.7)	25/43(58.1)	
**T3a**	11/93 (11.8)	26/170 (15.3)		5/19 (26.3)	18/55 (32.7)		5/14(35.7)	11/43(25.6)	
**T3b**	36/93 (38.7)	14/170 (8.2)		11/19 (57.9)	8/55 (14.5)		4/14(28.6)	7/43(16.3)	
**T4**	44/93 (47.4)	0/170		0/19	0/55		0/14	0/43	
**Bone metastasis**,** No. (%)**	55/93 (59.1)								
**MLN short-axis mean(cm)**	2.3								
**LNM number**	164								
**Biopsy findings**,** No. (%)**			0.043*			0.000*			0.000*
**GS 3+3**	0	7/170 (4.1)		0/19	3/55 (5.5)		0/14	0/43	
**GS 3+4**	13/93 (14.0)	21/170 (12.4)		2/19 (10.5)	15/55 (27.3)		0/14	11/43 (25.6)	
**GS 4+3**	29/93 (31.2)	71/170 (41.8)		1/19 (5)	16/55 (29.1)		1/14 (7.1)	20/43 (46.5)	
**GS≥4+4**	51/93 (54.8)	71/170 (41.8)		16/19 (84.2)	21/55 (38.2)		13/14 (93.9)	12/43 (27.8)	
**Surgical findings**,** No. (%)**						0.000*			0.000*
**GS 3+3**	Non	10/170 (5.8)		0/19	4/55 (7.3)		0/14	0/43	
**GS 3+4**	Non	22/170 (12.9)		2/19 (10.5)	14/55 (25.5)		0/14	11/43 (25.6)	
**GS 4+3**	Non	67/170 (39.4)		1/19 (5)	16/55 (29)		1/14 (7.1)	21/43 (48.8)	
**GS≥4+4**	Non	71/170 (41.9)		16/19 (84.2)	21/55 (8.2)		13/14 (93.9)	11/43 (25.6)	
**Pathological stage**,** No. (%)**						0.000*			0.000*
**T2**	Non	135/170 (79.4)		4/19(21.1)	29/55 (52.7)		6/14 (42.8)	23/43 (53.5)	
**T3a**	Non	21/170 (12.3)		2/19(10.5)	18/55 (32.7)		4/14 (28.6)	13/43 (30.2)	
**T3b**	Non	14/170 (8.2)		10/19 (52.7)	8/55 (14.5)		4/14 (28.6)	7/43 (16.3)	
**T4**	Non	0/170		3/19 (15.8)	0/42		0/14	0/43	

PLNM = pelvic lymph node metastasis; MLN = metastatic lymph node; PSA = prostate-specific antigen

*P value < 0.05

### Feature extraction and model construction

To ensure the stability of the features, two radiologists independently delineated the ROIs slice by slice on the MR images of 30 patients.

**Fig. 4:**
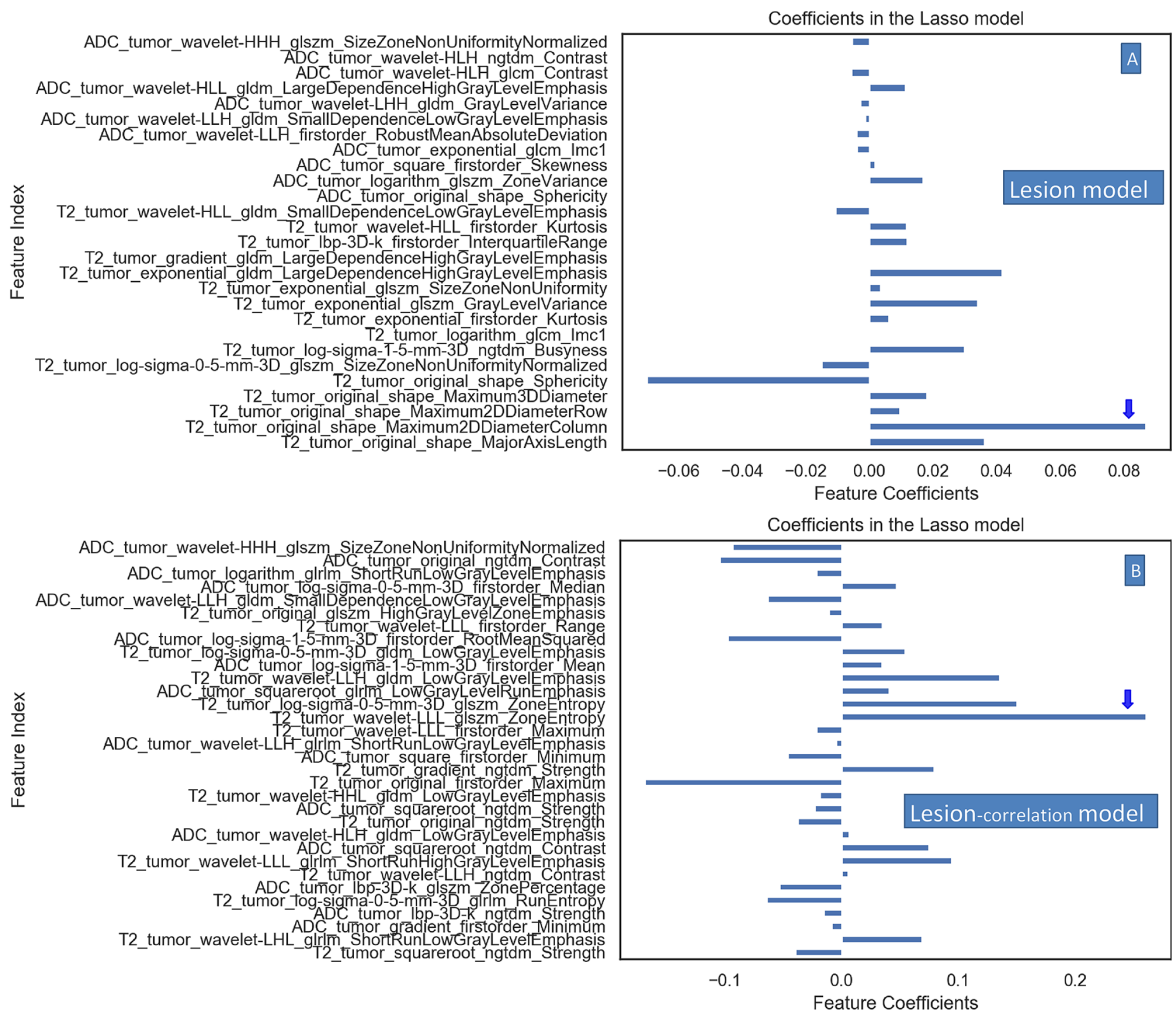
(**A**, **B**) shows the radiomic features and weights of the feature coefficients in the lesion model and lesion-correlation model, respectively. Figure 4 (**A**, **B**) shows that in the feature coefficient column, the right side indicates a positive correlation, and the left side indicates a negative correlation. The feature weights were generated by the lasso algorithm. Figure 3-A shows that among all the features (*n* = 27), “T2_tumor_original_shape_Maximum2ddiameter column” accounted for the highest weight coefficient and was positively correlated (blue arrow) in the Lesion model. Figure 3-B shows that among all the features (*n* = 32), “T2_tumor_wavelet_ LLL_glszm_ZoneEntropy” accounted for the highest weight coefficient and was positively correlated in the Lesion-correlation model (blue arrow). LASSO = absolute shrinkage and selection operator

Two groups of features (3748 features each) were extracted from VOI-1 and VOI-2. For the lesion model, the primary lesion RFs were screened by the t test and LASSO algorithm, and 27 features were ultimately selected, thereby establishing the lesion model. Additionally, ‘’T2_tumor_original_shape_Maximum2ddiametercolumn’’ had the highest weight coefficient and was positively correlated among all the features included in the lesion model (Fig. 4A). For the lesion-correlation model, the extracted features of the metastatic LNs and primary lesions were analyzed by Pearson correlation analysis algorithm, 280 features of the primary lesions were obtained, and then feature screening by the t test and LASSO algorithm was performed. Finally, 32 features were selected, thereby establishing the lesion-correlation model. Further, “T2_tumor_wavelet_LLL_glszm_ZoneEntropy’’ accounted for the highest weight coefficient and was positively correlated among all the features included in the lesion-correlation model (Fig. 4B).

### Model comparison and validation

**Fig. 5:**
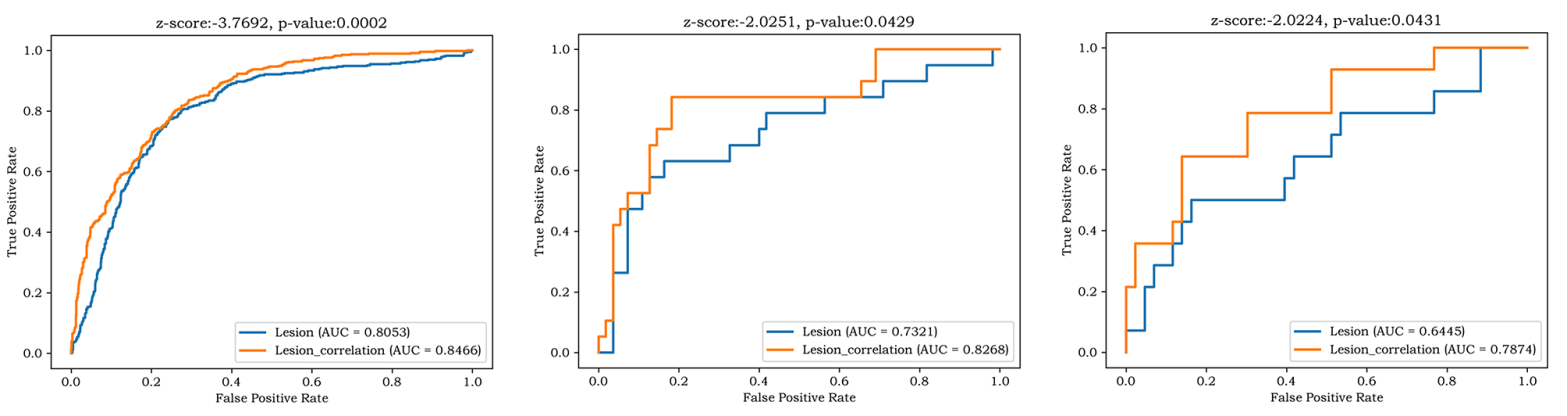
shows that the ROC curves, AUC and DeLong test of AUC in the lesion model and lesion-correlation model were 0.8053, 0.8466, and 0.0002 in the training groups, 0.7321, 0.8268, and 0.0429 in the internal validation group, and 0.6445, 0.7874, and 0.0431 in the external validation group, respectively. AUC = area under the receiver operating curve

Table 2 shows the AUC and 95% CI of the lesion model and lesion-correlation model in the training group [0.8053 (95% CI: 0.7876–0.8230); 0.8466 (95% CI: 0.8317–0.8615)], internal validation group [0.7321 (95% CI: 0.5854–0.8787);0.8268 (95% CI:0.7096–0.9440)], and external validation group [0.6445 (95% CI:0.4618–0.8273); 0.7874 (95% CI:0.6466–0.9281)], respectively; The DeLong test of the AUC values of the lesion model and lesion-correlation model in the training and two validation groups were 0.0002, 0.0429 and 0.0431, respectively. The ROC curve, AUC, accuracy, sensitivity, specificity and DeLong test of the two models in the training, internal validation and external validation groups are shown in the following chart (Table 2 ; Fig. 5).

## Discussion

LN metastatic lesions “flow” from primary lesions along lymphatic vessels and grow in the LNs. Although the growth environment differs from that of the primary lesions, the histological characteristics are the same, as demonstrated by the immunohistochemical examination results. Therefore, MLNs contain the cancer tissue derived from the primary lesions, which is more likely to reflect the metastatic characteristics of the primary lesions. Radiomics is an emerging research technology that reflects changes at the tumour tissue, cell and gene levels by mining the RFs of tumours [33, 34]. That is, the RFs of MLNs can reflect the metastatic characteristics of primary lesions. Therefore, it can be considered that a correlation analysis between the RFs of primary lesions and MLNs can better select the RFs of primary lesions reflecting metastatic characteristics. Unlike previous approaches focusing on the task of algorithm selection [11, 13, 35–37], in this study, the RFs of pelvic MLNs in PCa were extracted for the first time and used to effectively screen the RFs reflecting PLNM in primary lesions by the correlation analysis algorithm, optimizing the feature selection of the primary lesions. This study results indicated that the lesion-correlation model had some advantages over the lesion model in predicting PLNM, the DeLong test of their AUC values in the training and two validation groups were 0.0002, 0.0429 and 0.0431,respectively, and confirmed that the RFs of primary lesions that were more predictive of PLNM could be screened by correlation analysis with the RFs of metastatic LNs.

The problem of locating and delineating metastatic lymph nodes on MR images must be solved before using the RFs of pelvic MLNs and primary lesions for correlation analysis. For MLNs confirmed by PLND, a previous study reported that only 30% of PLNMs were identified by CT or MRI, because most MLNs were less than 8 mm [17, 38], and 83% of them were ≤ 5 mm and 50% were ≤ 3 mm [39]. Relevant literature has also confirmed that the volume of MLNs in PLND is generally small, and most of them are not detectable on conventional MRI [40, 41], which makes it difficult to locate and delineate MLNs on the images. To solve the difficulty, this study stipulated specific inclusion criteria for the PLNM-positive patients in the training group, i.e., LNs with abnormal signal intensity on bpMRI and those ≥ 1.5 cm in the short-axis diameter.

The premise of tumour metastasis is the aggressiveness of tumour cells and infiltrative growth in the surrounding stroma. The former reflects the attack of tumour cells on the surrounding tissues, such as the destruction of lymphatic vessels and blood vessels; and the latter reflects the growth of tumour cells in the surrounding tissues, resulting in local spread of the tumour. Regarding tumour aggressiveness, some studies have found that the homogeneity and entropy features from GLCM and GLRLM are significantly correlated with PCa aggressiveness and significantly differ between low- and intermediate/high-aggressive PCa, as defined by histopathology [42–44]. Our findings similarly showed that “T2_tumor_wavelet_LLL_glszm_ zone Entropy” had the highest weight coefficient among all the features included in the correlation model; however, in contrast with previous studies, our entropy feature was extracted from GLSZM. The main reason may be that most of our data are from patients with highly aggressive PCa, and all of them have PLNM, which is partially consistent with previous studies involving the prediction of tumour PLNM [13, 45]. These findings indicated that the entropy feature of GLSZM from T2WI could be used as an important marker to predict PLNM in PCa. Among all the features included in the lesion model, this study found that *“*T2_tumor_original_shape_ Maximum2d diameter column” had the highest weight coefficient, which described the regional feature of the internal tumour shape, and it also might reflect another feature of tumour metastasis, namely tumour infiltration, which mainly relates to the local spread of the tumour. Additionally, this study found that in both models, the weight coefficient of features ranked ahead were from T2WI, not from the ADC map (Fig. 4). We speculated that compared with the texture features from the ADC map, those from T2WI can better reflect the aggressiveness of tumors. In summary, these results indicated that the texture features obtained by correlation analysis with MLNs might be of greater value in predicting LNM and worthy of further exploration.

Our study had several limitations. First, the majority of pelvic MLNs in the training group were diagnosed by prostate MRI, a minority of which were confirmed by biopsy or therapy. Second, deep features of pelvic MLNs were not extracted, and a deep learning algorithm will be applied in future research. Third, the cases in this paper underwent imaging with two types of imaging devices (Siemens and Philips), which were inconsistent in manufacturer performance and scanning parameters.

## Conclusion

Radiomics allows for the extraction of a large number of RFs from VOIs. The selection of effective features will directly affect the performance of the prediction model. The correlation analysis algorithm with pelvic MLNs could be used to effectively screen the RFs of primary lesions that reflected the characteristics of PLNM, and improve the performance of the primary lesions in predicting PLNM and provide new insights into the feature selection process.

**Table 2:** The performance of the two models in the primary training and validation groups

	Models	AUC	95%CI	Accuracy	Sensitivity	Specificity	Delong-test
							p-value
**Training group**	Lesion radiomics	0.8053	[0.7876-0.8230]	0.7635	0.714	0.7906	0.0002
	Lesion-correlation radiomics	0.8466	[0.8317-0.8615]	0.7511	0.8344	0.7053	
**Internal validation group**	Lesion-radiomics	0.7321	[0.5854-0.8787]	0.6622	0.6842	0.6545	0.0429
	Lesion-correlation radiomics	0.8268	[0.7096-0.9440]	0.7838	0.8421	0.7636	
**External validation group**	Lesion-radiomics	0.6445	[0.4618-0.8273]	0.6491	0.5	0.6977	0.0431
	Lesion-correlation radiomics	0.7874	[0.6466-0.9281]	0.807	0.6429	0.8605	

AUC = area under the receiver operating curve; CI = confidence interval

### Electronic supplementary material

Below is the link to the electronic supplementary material.

Supplementary Material 1

Supplementary Material 2

Supplementary Material 3

Supplementary Material 4

Supplementary Material 5

## Data Availability

All data generated or analysed during this study are included in this published article/supplementary material, further inquiries can be directed to the corresponding author.

## Abbreviations

PCa: Prostate cancer
RF: Radiomic feature
PLNM: Pelvic lymph node metastasis
MLN: Metastasis lymph node
mpMRI: Multiparametric MRI
bpMRI: Biparametric MRI
RP: Radical prostatectomy
LASSO: Absolute shrinkage and selection operator
ePLND: Extended pelvic lymph node dissection
T2WI: T2-weighted imaging
DWI: Diffusion-weighted imaging
DCE: Dynamic contrast-enhanced
ADC: Apparent diffusion coefficient
VOI: Volume of interest
ROC: Receiver operating characteristic
AUC: Area under the curve
