# Supplementary figures and images for: Biparametric MRI of the prostate radiomics model for prediction of pelvic lymph node metastasis in prostate cancers : a two-centre study
Source: BMC Med Imaging. 2024 Jul 25;24:185. doi: 10.1186/s12880-024-01372-8 (PMC11271060; doi:10.1186/s12880-024-01372-8)

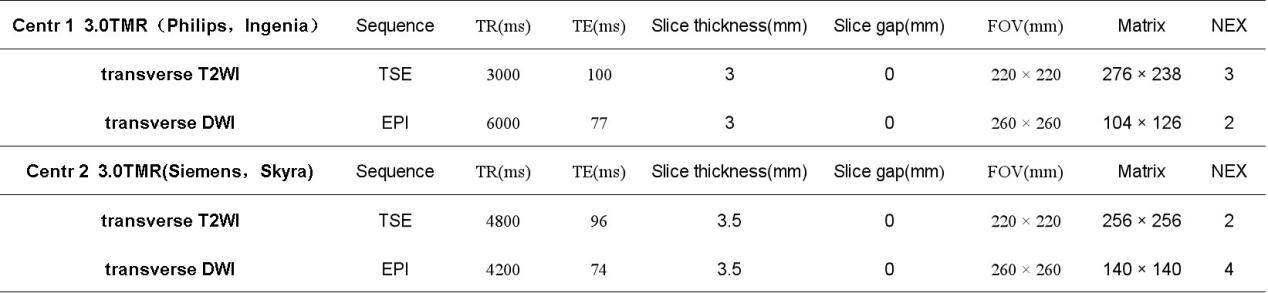


**Supplementary 1** shows the scan parameters and machine models of hospitals.

Supplement: Supplementary file 1 — Supplementary Material 1 [file 12880_2024_1372_MOESM1_ESM.docx]
