# Supplementary material for: Biparametric MRI of the prostate radiomics model for prediction of pelvic lymph node metastasis in prostate cancers : a two-centre study
Source: BMC Med Imaging. 2024 Jul 25;24:185. doi: 10.1186/s12880-024-01372-8 (PMC11271060; doi:10.1186/s12880-024-01372-8)
Supplement: Supplementary file 3 — Supplementary Material 3 [file 12880_2024_1372_MOESM3_ESM.docx]

**METhodological RadiomICs Score (METRICS): A quality scoring tool for radiomics research endorsed by EuSoMII**

**ELECTRONIC SUPPLEMENTARY MATERIAL**

**Supplementary file 3:** METRICS tool with explanations.

| **Categories** | **No.** | **Items** | **Weights** | **Score**^6^ |
| --- | --- | --- | --- | --- |
| Study Design | #1 | Adherence to radiomics and/or machine learning-specific checklists or guidelines  >>> Whether any guideline or checklist, e.g., CLEAR checklist, is used in designing and reporting, as appropriate for the study design (e.g., handcrafted radiomics or deep learning pipeline). | 0.0368 | 0.0368 |
|  | #2 | Eligibility criteria that describe a representative study population  >>> Whether inclusion and exclusion criteria are explicitly defined. These should lead to a representative study sample that matches the general population of interest for the study aim. | 0.0735 | 0.0735 |
|  | #3 | High-quality reference standard with a clear definition  >>> Whether the reference standard or outcome measure is representative of the current clinical practice and robust. Examples of high-quality reference standards are preferably histopathology, well-established clinical and genomic markers, the latest version of the prognostic tools, guideline-based follow-up or consensus-based expert opinions. Examples of poor quality reference standards are those based on qualitative image evaluation, images that are later used for feature extraction, or outdated versions of prognostic tools. | 0.0919 | 0.0919 |
| Imaging Data | #4 | Multi-center  >>> Whether more than one institution is involved as a diagnostic imaging data source for radiomics analysis. | 0.0438 | 0.0438 |
|  | #5 | Clinical translatability of the imaging data source for radiomics analysis  >>> Whether the source of the radiomics data is an imaging technique that reflects established standardization approaches, such as acquisition protocol guidelines (e.g., PI-RADS specifications). | 0.0292 | 0.0292 |
|  | #6 | Imaging protocol with acquisition parameters  >>> Whether the image acquisition protocol is clearly reported to ensure the replicability of the method. | 0.0438 | 0.0438 |
|  | #7 | The interval between imaging used and reference standard  >>> Whether the time interval between the diagnostic imaging exams (used as an input for the radiomics analysis) and the outcome measure/reference standard acquisition is appropriate to validate the presence or absence of target conditions of the radiomics analysis at the moment of the diagnostic imaging exams. | 0.0292 | 0.0292 |
| Segmentation^1^ | #8 | Transparent description of segmentation methodology  >>> Whether the rules or the method of the segmentation are defined (e.g., margin shrinkage, peri-tumoral sampling, details of segmentation regardless of whether manual, semi-automated or automated methods are used). In the case of DL-based radiomics, the segmentation can refer to the rough delineation with bounding boxes or cropping the image around a region of interest. | 0.0337 | 0.0337 |
|  | #9 | Formal evaluation of fully automated segmentation2  >>> If a segmentation technique that does not require any sort of human intervention is used, examples of the results should be presented and a formal assessment of its accuracy compared to domain expert annotations included in the study (e.g., DICE score or Jaccard index compared with a radiologist’s semantic annotation). Any intervention to the annotation in terms of volume or area should be considered as the use of a semi-automated segmentation technique. This item also applies to the use of segmentation models previously validated on other datasets. | 0.0225 | 0 |
|  | #10 | Test set segmentation masks produced by a single reader or automated tool  >>> Whether final segmentation in the test set is produced by a single reader (manually or with a semi-automated tool) or an entirely automated tool, to better reflect clinical practice. | 0.0112 | 0.0112 |
| Image Processing and Feature Extraction | #11 | Appropriate use of image preprocessing techniques with transparent description  >>> Whether preprocessing of the images is appropriately performed considering the imaging modality (e.g., gray level normalization for MRI, image registration in case of multiple contrasts or modalities) and feature extraction techniques (i.e., 2D or 3D) that are used. For instance, in the case of large slice thickness (e.g., ≥5 mm), extreme upsampling (e.g., 1 x 1 x 1 mm3) of the volume might be inappropriate. In such a case, 2D feature extraction could be preferable, ensuring in-plane isotropy of the pixels. On the other hand, achieving isotropic voxel values should be targeted in 3D feature extraction, to allow for texture feature rotational invariance. Also, whether gray level discretization parameters (bin width, along with resulting gray level range, or bin count) are described in full detail. Description of different image types used (e.g., original, filtered) should also be included (e.g., high and low pass filter combinations for wavelet decomposition, sigma values for Laplacian of Gaussian edge enhancement filtering). If the image window is fixed, it should be clarified. | 0.0622 | 0.0622 |
|  | #12 | Use of standardized feature extraction software^3^  >>> Whether a standardized software (e.g., compliant with IBSI) was used for feature extraction, including information on the version number. | 0.0311 | 0.0311 |
|  | #13 | Transparent reporting of feature extraction parameters, otherwise providing a default configuration statement  >>> Whether feature types (e.g., hand-crafted, deep features) and feature classes (for hand-crafted) are described. Also, if a default configuration statement is provided for the remaining feature extraction parameters. A file presenting the complete configuration of these settings should be included in the study materials (e.g., parameter file such as in YAML format, screenshot if a dedicated file for this is not available for the software). In the case of DL, neural network architecture along with all image operations should be described. | 0.0415 | 0.0415 |
| Feature Processing | #14 | Removal of non-robust features^4^  >>> Whether unstable features are removed via test-retest, reproducibility analysis by analysis of different segmentations, or stability analysis [i.e., image perturbations]. Instability may be due to random noise introduced by manual or even automated image segmentation or exposed in a scan-rescan setting. The specific methods used should be clearly presented, with specific results for each component in multi-step feature removal pipelines. | 0.0200 | 0.0200 |
|  | #15 | Removal of redundant features^4^  >>> Whether dimensionality is reduced by selecting the more informative features such as with algorithm-based feature selection (e.g., LASSO coefficients, Random Forest feature importance), univariate correlation, collinearity, or variance analysis. The specific methods used should be clearly presented, with specific results for each component in multi-step feature removal pipelines. | 0.0200 | 0.0200 |
|  | #16 | Appropriateness of dimensionality compared to data size^4^  >>> Whether the number of instances and features in the final training data set is appropriate according to the research question and modeling algorithm. This should be demonstrated by statistical means, empirically through consistency of performance in validation and testing, or based on previous evidence in the literature. | 0.0300 | 0.0300 |
|  | #17 | Robustness assessment of end-to-end deep learning pipelines5  >>> Whether DL pipeline consistency of performance has been assessed in a test-retest setting, for example by a scan-rescan approach, use of segmentations by different readers, or stability analysis [i.e., image perturbations]. The specific methods used should be clearly presented. | 0.0200 | 0 |
| Preparation for Modeling | #18 | Proper data partitioning process  >>> Whether the training-validation-test data split is done at the very beginning of the analysis pipeline, prior to any processing step. Data split should be random but reproducible (e.g., fixed random seed), preferably without altering outcome variable distribution in the test set (e.g., using a stratified data split). Moreover, the data split should be on the patient level, not the scan level (i.e., different scans of the same patient should be in the same set). Proper data partitioning should guarantee that all data processing (e.g., scaling, missing value imputation, oversampling or undersampling) is done blinded to the test set data. These techniques should be exclusively fitted on training (or development) data sets and then used to transform test data at the time of inference. If a single training-validation data split is not done and a resampling technique (e.g., cross-validation) is used instead, test data should always be handled separately from this. | 0.0599 | 0.0599 |
|  | #19 | Handling of confounding factors  >>> Whether potential confounding factors were analyzed, identified if present, and removed if necessary (e.g., if it has a strong influence on generalizability). These may include different distributions of patient characteristics (e.g., gender, lesion stage or grade) across sites or scanners. | 0.0300 | 0.0300 |
| Metrics and Comparison | #20 | Use of appropriate performance evaluation metrics for task  >>> Whether appropriate accuracy metrics are reported, such as AUC for Receiver Operating Characteristics (ROC) or Precision-Recall (PRC) curves and confusion matrix-derived accuracy metrics (e.g., specificity, sensitivity, precision, F1 score) for classification tasks; MSE, RMSE, and MAE for regression tasks. For classification tasks, the confusion matrix should always be included, to allow the calculation of additional metrics. If training a DL network, loss curves should be presented. | 0.0352 | 0.0352 |
|  | #21 | Consideration of uncertainty  >>> Whether uncertainty measures are included in the analysis, such as 95% confidence interval (CI), standard deviation (SD), or standard error (SE). Report on methodology to derive that distribution (ie. bootstrapping with replacement, etc). | 0.0234 | 0.0234 |
|  | #22 | Calibration assessment  >>> Whether the final model’s calibration is assessed. | 0.0176 | 0.0176 |
|  | #23 | Use of uni-parametric imaging or proof of its inferiority  >>> Use of a single imaging set (such as a single MRI sequence rather than multiple, or a single phase in a dynamic contrast-enhanced scan) should be preferred, as multi-parametric imaging may unnecessarily increase data dimensionality and risk of overfitting. Therefore, in the case of multi-parametric studies, uni-parametric evaluations should also be performed to justify the need for a multi-parametric approach by formally comparing their performance (e.g., DeLong’s or McNemar’s tests). This item is also intended to reward studies that experimentally justify the use of more complex models compared to simpler alternatives, in regard to input data type. | 0.0117 | 0.0117 |
|  | #24 | Comparison with a non-radiomic approach or proof of added clinical value  >>> Whether a non-radiomic method that is representative of the clinical practice is included in the analysis for comparison purposes. Non-radiomic methods might include semantic features, RADS or RECIST scoring, and simple volume or size evaluations. If no non-radiomics method is available, proof of improved diagnostic accuracy (e.g., improved performance of a radiologist assisted by the model’s output) or patient outcome (e.g., decision analysis, overall survival) should be provided. In any case, the comparison should be done with an appropriate statistical method to evaluate the added practical and clinical value of the model (e.g., DeLong’s test for AUC comparison, decision curve analysis for net benefit comparison, Net Reclassification Index). Furthermore, in case of multiple comparisons, multiple testing correction methods (e.g., Bonferroni) should be considered in order to reduce the false discovery rate provided that the statistical comparison is done with a frequentist approach (rather than Bayesian). | 0.0293 | 0 |
|  | #25 | Comparison with simple or classical statistical models  >>> Whether a comparison with a simple baseline reference model (such as a Zero Rules/No Information Rate classifier) was performed. Use of machine learning methods should be justified by proof of increased performance. In any case, the comparison should be done with an appropriate statistical method (e.g., DeLong’s test for AUC comparison, Net Reclassification Index). Furthermore, in case of multiple comparisons, multiple testing correction methods (e.g., Bonferroni, Benjamini–Hochberg, or Tukey) should be considered in order to reduce the false discovery rate provided that the statistical comparison is done with a frequentist approach (rather than Bayesian). | 0.0176 | 0.0176 |
| Testing | #26 | Internal testing  >>> Whether the model is tested on an independent data set that is sampled from the same source as the training and/or validation sets. | 0.0375 | 0.0375 |
|  | #27 | External testing  >>> Whether the model is tested with independent data from other institution(s). This also applies to the studies validating the previously published models trained at another institution. | 0.0749 | 0.0749 |
| Open Science | #28 | Data availability  >>> Whether any imaging, segmentation, clinical, or radiomics analysis data is shared with the public. | 0.0075 | 0 |
|  | #29 | Code availability  >>> Whether all scripts related to automatic segmentation and/or modeling are shared with the public. These should include clear instructions for their implementation (e.g., accompanying documentation, tutorials). | 0.0075 | 0 |
|  | #30 | Model availability  >>> Whether the final model is shared in the form of a raw model file or as a ready-to-use system. If automated segmentation was employed, the corresponding trained model should also be made available to allow replication. These should include clear instructions for their usage (e.g., accompanying documentation, tutorials). | 0.0075 | 0 |
| Total METRICS score (should be given as percentage) | | | | 91.32% |
| Quality category^7^ | | | | excellent |

^1^ Conditional for studies including region/volume of interest labeling. ^2^ Conditional for studies using fully automated segmentation. ^3^ Conditional for the hand-crafted radiomics. ^4^ Conditional for tabular data use. ^5^ Conditional on the use of end-to-end deep learning.^6^ Score is simply the weight if present and 0 otherwise. ^7^ Proposed total score categories: 0≤score<20%, “very low”; 20≤score<40%, “low”; 40≤score<60%, “moderate”; 60≤score<80%, “good”; and 80≤score≤100%, “excellent” quality.
