# Supplementary material for: Biparametric MRI of the prostate radiomics model for prediction of pelvic lymph node metastasis in prostate cancers : a two-centre study
Source: BMC Med Imaging. 2024 Jul 25;24:185. doi: 10.1186/s12880-024-01372-8 (PMC11271060; doi:10.1186/s12880-024-01372-8)
Supplement: Supplementary file 5 — Supplementary Material 5 [file 12880_2024_1372_MOESM5_ESM.docx]

Supplementary material (5)

To perform the grey-level normalization step, we set the parameters as follows:

interpolator:sitkBSpline

resampledPixelSpacing:[1，1，1]

According to the example in PyRadiomics, we conducted the setting value as following.:

interpolator: 'sitkBSpline'

resampledPixelSpacing: [1, 1, 1]

padDistance: 10

binWidth: 25

voxelArrayShift: 300

label: 1

normalize: true

normalizeScale: 100
